# The possibility of physicalism

**DOI:** 10.1590/S1980-57642011DN05040002

**Published:** 2011

**Authors:** Eduardo Giannetti

**Affiliations:** Insper – Instituto de Ensino e Pesquisa, São Paulo SP, Brazil.

**Keywords:** physicalism, mind-body relationship, mind-brain relationship, mental events

## Abstract

Modern science has undermined belief in countless imaginary causalities. What is the nature of the relation between mind and brain? Philosophers have debated the issue for millennia, but it is only in the last twenty years that empirical evidence has begun to uncover some of the secrets of this ancient riddle. This lecture explores the possiblity that advances in neuroscience will undermine and subvert our intuitive, mentalist understanding of the mind-body relationship. Recent findings in neuroscience seem to support the notions that (i) mental events are a subclass of neurophysiological events, and (ii) they are devoid of causal efficacy upon the workings of the brain. If physicalism is true then the belief in the causal potency of conscious thoughts and free will are bound to join company with countless other imaginary causalities exploded by the progress of science.

## Abstract (pt)

A ciência moderna tem minado a crença em inúmeras causalidades imaginárias. Qual é a natureza da relação entre mente e cérebro? Filósofos têm debatido a questão por milênios, mas apenas nos últimos 20 anos a evidência empírica começou a descobrir alguns dos segredos deste antigo enigma. Esta palestra explora a possibilidade de que os avanços na neurociência vão corroer e subverter a nossa compreensão intuitiva e mentalista da relação mente-corpo. Descobertas recentes da neurociência parecem apoiar as noções de que (i) os eventos mentais são uma subclasse de eventos neurofisiológicos, e (ii) que são desprovidos de eficácia causal sobre o funcionamento do cérebro. Se o fisicalismo é verdade, então as crenças na potência causal de pensamentos conscientes e no livre arbítrio estão destinadas a fazer companhia a inúmeras outras causalidades imaginárias que foram destruídas pelo progresso da ciência.

I - ***THE MIND-BRAIN PROBLEM.*** **PUT LIKE THIS, IT** may sound as a technical or abstruse question, divorced from the realities of life - the kind of issue best left to experts in white coats, philosophers or neurologists. Nothing could be further from the truth. For the fact is that each and every one of us, no matter how unconcerned, holds a set of spontaneous beliefs on the nature of the relationship between our body - brain included - and our mental world - "the sheer chaotic, tropical luxuriance of the inner life", to quote American philosopher Thomas Nagel.^1^

The first and decisive steps towards fixing these beliefs take place in early infancy. When a small child begins to hone her visual attention and to realize her body is separate from the rest of the world; when she becomes aware that some movements of her arms and legs *stem from her* while other movements - such as when she is held up or pushed around - are produced by *external* forces; when she realizes that whenever she nips her brother's arm he squeals while she feels nothing, whereas every time he pinches her arm it hurts and she hollers while he merely smirks; when this child realizes that certain things are under the sway of her will while others oddly aren't, she is in fact learning to walk on her own two feet in an unfolding mental world - she is finding her bearings in a rich and intricate network of beliefs about the mind-body relationship which will become part of her inner life for the rest of her days. It is a rite of passage we all go through.

At the other end of the life's arch distinct features of the mind-brain interlace come to the fore. An elder who becomes dependent on sleeping pills due to insomnia and finds that his short-term memory evaporates while long-forgotten memories of the remote past erupt vividly to mind; a man who finds that his libido wanes as the years go by, giving way to an unusual sense of peace; an elderly woman who, despite trying, has never felt as old in her mind as the mirror portrays she is; a terminally-ill patient who ponders as never before about what will become of him, if anything, when his time is up - all of these individuals, whether aware of it or not, are dealing with themes and concerns regarding the mind-brain relationship. Many of us will experience - if not have already done so - something similar.

From the pre-linguistic world of a toddler to the typical delusions and regressions of senility, the thread of mental life comes full circle. In the interval spanning these extremes, and however crude and simple their conceptions, all human beings have a modicum of the spontaneous philosopher or novice metaphysicist in them - they carry a network of beliefs and notions about what it means to be a person endowed with one's own conscience and will, and about our place as a species in the universe about us.

What kind of creature is man? How do we differ from other living creatures? Is there some kind of a widely-shared set of beliefs or common-sense regarding this peculiar amalgam of bodily and mental realities which we experience in our lives?

**II - PERSONAL IDIOSYNCRASIES AND VARIATIONS** aside, I believe that a common understanding does exist - a pre-reflective and reasonably robust shared view, albeit mostly latent, concerning the mind-body relationship. This network of spontaneous beliefs, acquired for the most part in early childhood, is grounded on *two pillars*.

The *first pillar* is the simple observation that there appears to be two different types of event taking place in the world: on the one hand, there are the events belonging to the external physical world perceived by our senses; and, on the other, there are all the events that belong to the realm of our mental world - the plethora of states and occurences succeeding each other in the inner life.

The events of the physical world are those which can be externally observed and empirically examined, although in some cases only by means of sophisticated devices such as microscopes, radars and tomographs. For instance, if you hear a loud bang or smell the perfumed aroma of jasmine, this produces observable and measurable changes in millions of nerve cells in the brain. The sound waves created by the bang, the volatile chemical elements associated to the jasmine smell, and the corresponding states of the brain, all belong to this physical world.

For events of the mental world, however, it is not like this. Everything going on in the mind - our thoughts and memories, moods and feelings, desires and sensations - are part of the experience of the individual and are entirely closed and hidden from outside view, although we can try to communicate them through words or other forms of expression.

What takes place in your inner experience when, for example, you are startled by a sudden bang or delighted at the smell of jasmine shall never be seen or felt, perceived or smelled by anyone else except you, no matter how sophisticated future research techniques for accurate imaging of complex neural alterations produced by what our senses transmit to the brain may become. Deep sleep dreaming is a mental event second to none - a manifestation of mental experience at its purest. (Woody Allen's lament - "My one regret in life is that I am not someone else" - in fact assumes what we can never actually get to know, that is, *what it feels like to be somebody else*; I suspect that, had he been given that chance, and provided he could still recall how it felt to be himself, he would quickly come to regret his decision and wish to switch back to his familiar self.)

Attribution of mental experience to real world objects - from animals and other living creatures to natural phenomena and inanimate entities - is highly variable. While a pure solipsist believes that he is the only being endowed with conscious life in the whole universe, advocates of panpsychism consider that nothing in the world, not even rocks, waterfalls or bacteria, is devoid of some degree of inner and latent mental experience, no matter how incipient and rudimentary it may be compared to what we experience in ourselves.

Despite the fact that beliefs held about the existence of souls, spirits and wills in nature were rather different in ancient times, it is reasonable to assume that most people in the modern world lie somewhere midway along the spectrum between the extremes of pure solipsism and radical panpsychism. The difference in attribution of inner experience to other creatures helps understand why most of us find it more disturbing to decapitate a chicken than to slice a lizard or squash a cockroach under foot.

The *second pillar* underpinning our shared understanding of life and ourselves is the belief that both the physical and the mental worlds are not impervious to one other but rather constantly interact. What takes place within my body - brain, torso and limbs - influences what goes on in my mental world and likewise, the choices I make - what I think and consciously decide to do - influences the visible movements of my body. The physical and mental realms comprise the same reality, and akin to a two-way street, the causal traffic between them runs both ways. They are both open in principle to the influence and actions of the other.

If I take an analgesic, the pain is relieved; if I remember to pay the dentist a visit, I physically go to the dental surgery. If I smoke a cigarette, I'll become more alert and lively; but when I think about the harm caused by nicotine I throw the pack out.

**III - OUR SPONTANEOUS BELIEF IN THE CAUSAL** power of the mind over physical reality comes strikingly to the fore in an experience in which we all partake - *rooting for something*. After all, what does it mean to yearn and root for something to happen? What goes on in our minds when we are in the midst of rooting for a particular outcome - when watching a football match for example?

The game played out on the pitch is the same for all: the final score is as solid as granite. But when it comes to the *game experienced* things change rather. What is the secret of the passion which overtakes us in the agony of supporting and yearning with a pounding heart? What mental processes and surges of the most singular subjectivity are not filtered by the eyes and minds riveted on each pass of the ball on the TV screen?

The soul of the supporter finds itself overwhelmed by the remotest kind of metaphysics. To *root for* involves the feeling of digging deep into our wishes and desires. It gives expression to the mental process of placing yourself and your emotions on the pitch as if they were intertwined with the bodies of the athletes and the whimful trajectories of the ball. The facial grimaces and gestures of the supporters are just an outward sign of the inner gymnastics which consumes them.

But it doesn't stop there. The subjective contortionism of supporting is intertwined to a tacit belief deeply ingrained in the act of yearning and rooting for something - a magical way of thinking and feeling that spreads in the mind with the vigour of a rampant shrub. Rooting for something is to give in to the primary and overwhelming belief that the contortions that stir and devour the soul can somehow *twist* the course of events in the desired direction. The cathartic explosion of the goal - or of a saved penalty - bears out the joy of confirming the sheer strength of one's subjective will.

Supporting is somewhat analogous to praying, only without ceremony or intermediaries. In praying, the devotee concentrates and opens up a channel of dialogue through language - she addresses the saint or deity of choice, urging them to intercede in her favor. Promises and sacrifices can facilitate the deal, but the efficacy of the prayer is not based merely on the raw will of the devotee. Success depends on a favorable decree by the authority in charge.

Naturally, the supporter may also pray and make promises, but in the heat of the moment he goes straight to the point. It is not something deliberate or that can be freely chosen or avoided; it is rather an involuntary process of the most ancient origin which takes us to a mythical world where our desires and emotions enjoy *causal power* over the unfolding and unpredictable course of events.

The supporter's inner bench of players takes to the pitch, deflects the threatening kick, blocks the pass, crosses the corner kick, heads the ball, and exults at the very moment his omnipotence is vindicated in the catharsis of the goal. A web of dreads and desires, hopes and reliefs join every strike and follow every twist of the ball. No wonder an avid supporter finds himself spent by the end of the match.

Deep down the faith of a supporter is the belief that we can master and twist the natural course of things - *coerce the future* - through the naked force of our will. The world, cries in silence the supporter's soul, is not deaf or impervious to my wishes; destiny is amenable to my overpowering will. (How often in my childhood, when games were not yet broadcast live on TV, did I root and enthuse intensely upon seeing games long finished, but whose final score I was as yet unaware?)

Supporters in competitive sports are of course members of an extended family. We root for the dear ones convalescing from illness; for the plane to beat the turbulence; for the weather to improve; for the goodies to win and the baddies to fail; for the telephone to ring or e-mail to arrive. Faced with an open-ended future with an uncertain outcome, the human animal does not give in to its own impotence and cosmic abandonment. Like an addicted gambler, he believes in his wishes as the causes of real effects and bets what he can in the magic roulette of his will: *when I twist inside, the world twists along*. The psychology of yearning and rooting for is a scandal of reason - a remnant of the most archaic animist faith surviving within us all.

**IV - OUR SPONTANEOUS NOTIONS ABOUT THE** mind-brain relationship reflect what we believe in. However, not all we believe, no matter how seemingly obvious or familiar, is worthy of belief. The natural propensity to believe in something is not a valid argument for that belief, just as the strength of a conviction is no guarantee of its veracity.

For one thing are the ideas and beliefs that since childhood have cluttered together like inherited furniture in our self-understanding; but quite another is the attempt to put the house in order, that is, to critically examine these ideas and beliefs, and to decide what to keep and what to discard. It is this house-keeping activity of reappraising our spontaneous beliefs about the mind-brain relationship that turns it into a philosophical problem.

All we know about the world and ourselves is the product of our minds. But what lies behind the workings of the mind? How does mental activity, which encompasses all we feel, think and dream, come about and how is it produced? What is the origin of the lush fauna and flora of our consciousness and inner life?

Speculation on the sources of mental activity goes a long way back. In the ancient world, there used to prevail the belief that not all that went on in our minds was produced by the body. Nightime dreams and hallucinations; sudden inspirations and the trance of lovers; the overwhelming passions and the audacity of heroic deeds were all regarded and experienced as manifestations or interventions of extranatural forces - such as the invasion of men's spirits by some divine or demonic entity (hence the term *enthusiasm*, whose root is the Greek word *enthous*: "taken or possessed by a god").

The notion that the body is the seat of the mind and responsible for its workings underwent numerous turns, alleys and byways before finally reaching the brain. Among the ancient Greeks, for instance, and according to popular belief, it was the liver that housed our emotions and desires, as well as mood swings. Remnants of this concept survive in our language: the term *melancholy* derives from the Greek words *melas*: "black" + *khole*: "bile" - the overproduction of bile secreted by the liver was believed to be the underlying cause of somber and depressive states of the mind.

Aristotle, for his part, considered the heart - and not the brain - as the organ responsible for emotions and the control of bodily movement; evidence of this, he claimed, was the fact that headless chickens could still run around for a while; the main function of the brain was as an organ for cooling the blood.

The discovery that the brain was the site of the mind - the place where all the events of our mental life are produced - was made in the 5th century BC by Greek physicians of the Hippocratic school. The passage in which Hippocrates outlined the role of the brain in human existence and refuted the prevailing belief that epilepsy was a "sacred disease" or some form of divine punishment, is worthy of note - the serene lucidity of the statement defeats the passage of time:

*It ought to be generally known that the source of our pleasure, merriment, laughter and amusement, as of our grief, pain, anxiety and tears, is none other than the brain. It is specially the organ that enables us to think, see and hear, and to distinguish the ugly and the beautiful, the bad and the good, pleasant and unpleasant. [...] It is the brain too which is the seat of madness and delirium, of the fears and frights which assail us, often by night, but sometimes even by day; it is there where lies the cause of insomnia and sleep-walking, of thoughts that will not come, forgotten duties and eccentricities. All such things result from an unhealthy condition of the brain; it may be warmer than it should be, or it may be colder, or moister or drier, or in any other abnormal state.*^2^

What Hippocrates couldn't have imagined is that some 2500 years after his insight it would be the fervent search for the sacred and for religious salvation that would be explained as "the result of a type of epilepsy in the so-called temporal area of the brain", as proposed by the biologist Francis Crick, co-discoverer of DNA.^3^

"People with this type of epilepsy", suggests Crick, "often tend to display exaggerated religious behavior; a historical figure such as St. Paul was almost certainly an epileptic and, more recently, Dostoievski was surely one. Many experiments are underway investigating the possibility of inducing religious experiences through excitation of the brain".

If Freud shocked pious souls in his time upon suggesting in *Totem and tabu*, that religion was a mild form of obsessive neurosis, Crick went a step further: the religious experience boils down to a bout of convulsive excitement occuring in varying degrees in individuals whose brains are subject to abnormal electrical discharges in the temporal lobe.

Notably, Crick's hypothesis is consistent with the observation of several philosophers dedicated to the study of religious temperament, including Nietzsche: "In the wake of repentance and redeption training we find tremendous epileptic epidemics, the greatest known to history, such as the St. Vitus' and St. John's dances of the Middle Ages";^4^ William James: "Religious geniuses have often shown symptoms of nervous instability [...] medical materialism finishes up St. Paul by calling his vision on the road to Damascus a discharging lesion of the occipital cortex, he being an epileptic";^5^ and E. M. Cioran: "Most subverters, visionaries, and saviors have been either epileptics or dyspeptics".^6^

While for the ancient Greeks (against whom Hippocrates objected) epilepsy was seen as a sacred disease, for today's neuroscience it is the manifestations of religious fervor, along with its transports, trances and hallucinations, that is understood as the by-product of an epileptic syndrome.

**V - HOW SHOULD REALITY, AND WHAT HAPPENS IN** it, be understood? Our spontaneous apprehension of things and of what goes on in the world is gleaned through the five senses. But to what degree is that which our senses convey actually trustworthy? To what extent do these sensations and perceptions of which we have immediate awareness correspond to the reality of *how things are* as opposed to the way they *appear to be*? Where does neuroscience stand regarding the nature of the mind-brain relationship?

Nothing arises out of nothing. There is a crucial difference between what exists objectively in the world, independently of us, and what is only subjective, that is, due to the effects of the world on our senses and our mind.

The key step was to put our natural senses and perceptions of things to the test. Scientific analysis has gradually shown that our spontaneous apprehensions result from a complex process of interaction between external phenomena, our perceptive apparatus and our nervous system; the aim is to offer a detailed account of their physical basis and of what underlies our mental experiences of different sights, sounds, colors, touch, tastes and smells of things.

The upshot of this investigation is that the world we are familiar with, and in which our subjective life takes its course, differs radically from the world as it really is. Upon examining our sensory experiences from the outside in, it became apparent that the things we apprehend are being processed, translated and re-codified by us, thus generating sensations and mental states which stand to the objective reality of the world more or less as, say, the *names of things* stand to the *things themselves*.

Two examples help illustrate the point. The first is the sensation of tickles. Imagine that someone gently rubs a feather on the sole of your foot and that this provokes in you a tingling sensation of tickles. What is this? What and where are the tickles - in the feather, the sole or the contact between them?

In fact, the tickling sensation is confined to the mental world or inner experience of the feeling subject. What actually exists is a light friction between the atoms of the feather and the atoms of the skin, creating a flow of atoms which run along the nerve connecting the skin's surface to the nervous system, and ultimately producing a tiny agitation of atoms in the brain which linguistic convention has named as *tickles* (an intriguing fact recently unearthed by neurophysiology is that, whereas we feel tickles produced by another, especially unexpectedly, we never manage to tickle ourselves).

The best evidence, however, comes from experiments carried out on the brain of patients submitted to neurosurgery. Since the encephalic matter - the spongy tissue receiving and processing sensations from all over the body - is itself insensitive to pain, intracranial experiments can be conducted with the patient awake, that is, able to report what is passing through their mind during the procedure, with just a local anesthetic applied to the scalp and enveloping membranes of the brain.

When a mild electrical pulse is applied directly to the surface of the somatosensorial cortex - the area of the brain which receives sensory messages from all parts of the body - the patients feels the conscious sensation that something is being applied to the skin, like a pinprick or sandpaper being rubbed on a particular point of their arm, stomach or other region of the body; when the electrode is moved across the surface of the cortex, the subjective and illusory site of the sensation associated to the stimuli shifts accordingly, specifically to the body part that transmits sensory information to the point of the cortex being stimulated.

The sensory and motor cortices contain a kind of "map" which represents the entire topology of the body. All the sensations coming from some point of the organism, the nose or knee for instance, can be generated by means of direct stimulation to the cortex, without the nose or knee being touched in any way.

When we cut our finger or receive a caress, the sensation of pain or pleasure we feel does not really occur at the site of the wound or touch, but at the point of the brain which processes the nerve messages coming from these areas. The illusion that the pain or pleasure are located in the affected part of the body, rather than the corresponding point of the somatosensorial cortex, only occurs because the brain, besides receiving the relevant information, projects back to the specific site of the wound or pleasure the evolutionarily relevant message alerting that some kind of injury to be avoided or pleasure to be had is taking place at that point.

The brain foils the mind. Electrical stimulation of other regions of the brain is able to produce not only involuntary muscular movements in the corresponding limbs, but also visual, olfactory and auditory sensations, hallucinations and even acutely vivid recall of faces, melodies, scenes and childhood experiences.

As far as the patient's subjective experience is concerned, all this occurs as if it were something actually happening whereas in reality we know that it amounts to nothing but the after-effects of an astute and artificially produced scientific stratagem - the artifacts of the manipulation of an electrode applied to the exposed brain of a patient in a surgical theater. Atoms in movement.

**VI - LET US NOW TURN TO THE RELATION BETWEEN** thought and action. What could be more familiar than the obvious link, at least in our mind's eye, between a conscious decision to act on the one hand, and its fulfilment as an action in the real world on the other? It as simple as "I want, therefore I do!". And yet, upon closer scrutiny of the nature of this causal connection between thought and action, in light of all we know about the world, it would be hard to conceive something less obvious, less clear or less intelligible.

The first step is to unravel the relationship between conscious will on the one hand, and bodily action on the other. What is the nature of and the mechanism behind this most peculiar of links?

It's a plain fact of life: a striking *duality* pervades the human condition. Virtually all that occurs within our organism - a myriad of metabolic processes essential for survival - are entirely cut off from the control of our conscious will. The heart beats, the blood circulates, and food is digested; in response to the appropriate stimulus, the liver secretes bile, the pores of the skin produce sweat, and the suprarenal glands, adrenalin. The list could go indefinitely on.

How does all this work? The brain structure responsible for the majority of the automatic processes of control and adjustment in the organism is known as the hypothalamus (Greek *hipo*: "under" + *thalamus*: the term designating each of the two masses of grey matter in the forebrain to which an intricate network of branched nerve fibers converges). It weighs no more than four grams and belongs to the limbic system (a generic term designating a more primitive region of the brain in evolutionary terms, associated to visceral sensations and the processing of emotions). Although tiny in size, the hypothalamus house a complex bundle of fibers and nerve cells rendering it the densest and most highly connected organ in the entire brain.

The striking thing, however, is that when we pass from the internal metabolism of the body to our actions in the external world, this reality seems to change radically. And at this point the *duality* comes in. For as we can easily find for ourselves, a significant subset of the muscles in our body, such as those governing the hands, tongue, arms and legs seem to respond to our conscious will and commands, and to have quite a different relationship with the mind.

If I decide to activate my tear glands right now, I will find myself unable to do so (a seasoned actress may have more success); but if I decide to blink or raise my eyebrows, they obey without hesitation. A political prisoner may choose to go on a hunger strike as a way of protesting, even though no effort of his conscious will would enable him to block hunger pangs or prevent food, once ingested, from being duly digested and assimilated.

The duality is there, but how can it be explained? Several questions arise. A first, scientific question, not yet fully elucidated, is: *why does conscious will exist at all?* If our inner metabolism is able to take care of itself, monitoring, reacting and adjusting autonomously to a seemingly infinite number of demands from the body, then why isn't all like this? In what manner and precisely why did the boundaries between closed mechanisms on the one side, and processes which appear to be open and receptive to the commands of our conscious will on the other, gradually become demarcated in the evolutionary process?

Vegetables are immobile; animals move in space. The more rudimentary the nervous system of an animal, the narrower its range motor responses and repertoire to deal with novel situations. What differentiates the human animal in this regard is the complexity and sophistication of their neurophysiological system for controlling movements of the body.

Our range of motor responses runs from the simple automatic reflex - withdrawing our hand from fire in a split second or scratching an itch while asleep - to the most demanding and subtle use of the fingers - a delicate surgery or a piano solo.

Fine control of our voluntary muscles is a complex operation which involves the synchronized action of several different areas of the brain, most notably the motor cortex: a recent development in evolutionary terms and relatively large in the human species. Electrical stimulation of specific points of the motor cortex produces movements in the corresponding muscles without the person intending or deciding to perform them.

The broadening of the motor response repertoire in the face of threats and opportunities which the world presents makes sound evolutionary sense. This enhanced ability endowed our species with an unique and highly useful flexibility of action in practical life; it empowered them to make other creatures and natural processes, starting with the domestication of plants and animals, to work on their behalf.

Take, for example, the interplay between a horse and its rider. No horse is born ready - taming is needed. Its friskiness must be "broken", that is, submitted to the will of the rider by means of conditioning and exercising. But when the horse is trained and ready, what have we got? The horse's muscles respond like a well-oiled machine to the commands given by the voluntary muscles of the rider through use of the reins, spurs, whip and sound prompts. Two bodies wonderfully attuned - one is the natural extension of the other.

In practice, the motor system of the rider co-opted the motor system of the horse in much the same way our voluntary muscles are co-opted by one's motor cortex and become, as a rule, docile and compliant to our commands. The control which humans exert over external nature is no more than a continuation by other means of the control that some parts of our own bodies manage to exert over some others.

The difference in the case of the horse, however, is that the control is exerted by means of the sensitive apparatus of the animal - a touch of the reins, the spurs' pricking, the rider's voice - whereas in our case commands are relayed via electrical pulses - atoms electrically charged with sodium and potassium - which connect the cells and synapses of the brain to the relevant muscles via the nerve filaments spread out in the body. All in a fraction of a second.

This divide is real. The physiology of a muscle such as the heart, with its systoles and diastoles, is not to be confounded with that of the voluntary muscles of the body. Up to this point we have remained in the realm of science, with its methods, hypotheses and empirical evidence.

**VII - THE PHILOSOPHICAL QUESTION RAISED BY** this duality is: what is the nature of such divide? Is there a radical ontological difference - a leap of absolute discontinuity - between all that takes place in the internal metabolism of the body and that which seems to happen whenever we decide to move our limbs to act in the external world? What actually lies behind this odd duality?

The mentalist view, grounded on our intuitive psychology, holds there is a radical gulf separating the two processes: while one is purely automatic, given and rigidly determined by the blind laws of nature, the other is a human prerogative, founded on a system of muscular control in which the mind is sovereign. Conscious will - a mental state - drives and marshals the relevant neurologic processes, while these in turn make the body's cavalry jump and trot.

But does that really account for the way things are? What is actually happening? The physicalist view questions our intuitive psychology and proposes an essentially different take on the true nature of the divide.

If mental states affect bodily states then the only point at which effective contact becomes reality is the causal link between an act of will and its fulfilment as an action in the world. But what occurs when I think and decide, let's say, to *lift a finger*?

Thanks to new techniques of monitoring and visualizing cerebral activity in real time, it is known that something observable happens at the exact moment in which the decision is taken - a phenomenon involving chemical and electrical alterations in the hundreds of millions of nerve cells making up the brain. But what in fact is the relationship between the mental and private event, that is, my intentional decision to lift a finger, and the cerebral and observable event, which is the unique microscopic configuration of the complex neural network correlated to this act? As La Rochefoucauld once put it: "Man often thinks he is in control when he is being controlled".^7^

A voluntary act involves the intention to act followed by the corresponding muscular action. In the time lapse between the intention and performance of the act, a ramping up of neural activity takes place in the brain areas responsible for the motor control of the activated muscles. None of this would surprise a mentalist. The critical point is what ensues. And what of the intention to act? Where does it come from? How does the conscious will to lift one's finger emerge in the mind?

As the American neuroscientist Benjamin Libet has empirically established, the astonishing fact is that the surge of neural activity - the physical event in the brain - *precedes* not only the activation of the relevant muscles but also the mental event itself, that is, it starts before the very consciousness of the decision to act.

An intention which we become aware of has an underlying mechanism and unfolds in time. The electroencephalic register of what occurs when I decide to lift a finger reveals that the neurological process of the act commences around three tenths of a second *before* I become aware of my intention to carry it out. In other words, it is as if my brain knew before me what I was just about to do, and not only had the grace of forewarning me of the decision, but also to make it coincide with the gratifying illusion that it was my conscious will that is in the driving seat and deciding to perform it.

As so much in the realm of neurons and synapses, the time lag may seem negligible. But it isn't - it is real, measurable and has practical and philosophical implications. The process that culminates in an apparently voluntary action starts out in a pre-conscious way in the brain, before the intention to act pops out in the mind's mirror.

A study in neuroeconomics using magnetic resonance imaging shows that faced with a buying decision, two areas of the brain perform a tug-of-war for controlling the outcome. On one side there is the *nucleus accumbens*, with its dopamine receptors always at the ready when a rewarding opportunity arises; while on the other there is the *insula* (Latin term for "island"), a region of the cortex associated with sensations of discomfort and unpleasant feelings such as those caused by a bad smell, insults or shelling out cash.

The surprising fact is that, by observing the degree of activation of the two neural circuits involved, the researchers are able to predict seconds in advance whether a potential buyer will purchase the good. While the customer vacillates over the decision to stump up the money, the brain has already made up its mind.

In the physicalist view, therefore, our mental experiences do not arise out of nowhere but are the product of the activity of neurons and synapses in the brain; they are not born ready but evolve in time. Human self-knowledge is shallow. The link between thought and action hides a complex objective reality to which we aren't introspectively privy. Our conscious and unconscious life and our actions in the world are the culmination of a vast and intricate neural activity which unfurls underneath the threshold of awareness but which science is steadily unveiling, measuring and elucidating.

If that's the case, then it is not the conscious will that activates the motor cortex and produces action but rather the activity of the nerve cells in certain areas of the brain which activates the motor cortex and muscles while at the same giving rise to the subjective sensation that it is the conscious will to act that triggers the action.

**VIII - FROM LIGHTENING TO THE FLIGHT OF THE** dragon fly, all that occurs in the physical world is amenable to be explained according to physical laws and principles. The scientific approach has shown that there is no need to seek for or invoke extra-physical variables - such as spirits, hidden souls, wills, psychic beings, demons or divine intervention - in order to grasp the phenomena of the natural world. The physical world is self-contained, that is, it holds within itself all that is needed to understand and account for what transpires in it.

It would be odd, to say the least, to conceive that human beings of flesh and blood, the fruit of two fusing gametes, do not fully belong to this world. *Nature does not make leaps.* But if everything that exists in the natural world - to which our organism belongs and where our lives are played out - can be fully accounted for by physical variables, then why should it be any different with us?

Although the human brain is an extraordinarily complex organ - the most intricate and intriguing known to us - this doesn't make it a kind of black-box: a supernatural organ driven by otherworldly forces, exempt from the natural laws of cause and effect or from the relations of time and space prevaing in the rest of nature.

Yet if our cells and organism (brain included) are physical entities which are born, grow and move in physical space-time, as occurs with all living creatures on Earth, then there is no need to resort to any extra-physical variable, such as our thoughts, intentions and conscious will, to explain our existence and actions in the world. It follows that a thoroughly scientific understanding of *Homo sapiens*, grounded on the search for clear, intelligible results subject to public scrutiny, rules out referring to mental states when what is at stake is the elucidation of what makes us the way we are and act the way we act.

Neuroscience is no exception. As American neuroscientist Roger Sperry has put it, speaking for his professional colleagues, "the conviction held by most brain researchers - up to some 99.9 percent of us, I suppose - [is] that conscious mental forces can be safely ignored, insofar as the objective, scientific study of the brain is concerned".^8^

There is no denial of the reality of consciousness or mental events: what is being rejected is their use as valid principles of explanation. There is no denial of the important lacunae still remaining in the scientific study of the mind-brain relationship. Those seeking to get up to speed with the results achieved to date will soon find themselves agreeing with the American biochemist Julius Axelrod, when he says that "the brain's electrochemical language is as rich and subtle as that of Shakespeare - and we are just beginning to learn our ABCs".^9^

Research hasn't as yet managed to crack open and elucidate how the brain's physiological processes actually relate to our mental, subjective experiences. Discovering the key that deciphers this hieroglyph and reveals the exact translation of one code into the alphabet of the other is the holy grail of neuroscience.

But whatever the precise answer may be, the crucial question remains: what is the *direction of causality*, if any, between mind and brain? Every one of our mental experiences, conscious or otherwise, seems to correspond to a well-defined and particular configuration of the brain. Who is driving who? Is there, in fact, "a driver"?

That changes in the brain's anatomy and chemistry affect our states of conscience is quite plain: nobody need excise the hippocampus or take LSD to prove this; a cup of coffee or an analgesic would suffice.

And what about the other way around? How would it be to go from a mental state - say, a subjective sensation such as "I'm hungry" - and ascertain how this affects the brain and ensuing actions? How can a mental event - something I become aware of upon thinking about what is going through my mind - objectively control or affect the observable and measurable activity of neurons, synapses and electrochemical flows in my brain?

Just try to imagine it. First, how did the sensation come into being? Clearly, it did not arise out of thin air; subjective hunger most likely reflects a condition of need of the cellular tissues which was then transmitted to the nervous system and finally climbed the ramp of conscience ("I'm feeling hungry"). And then what? In the natural order of things, the feeling of hunger is followed by another mental state, which is the intention to act ("I've got to eat"), and the practical action of famished nature in search of satiation (lunch). Bur what is actually going on here?

A mentalist would say: mental events, in this case the feeling of hunger and the intention of eating, produce from top down the physiological processes of the brain as well as priming the motor cortex to activate the muscles of the body toward taking action to satiate hunger.

Note: what we have here is immaterial psychic entities rattling neurons and firing off synapses all over the place in an inscrutable ballet until the firing of electrochemical pulses acts on the branched nerve fibers of the body stimulating the muscles to dance. Choreography of rare and ineffable subtlety.

However well meaning, the notion that something of this sort might in fact be taking place seems so obscure and incongruent with all that is known about the natural laws governing the world that the only solution is to resort to Tertullian's response when faced with the mysteries of faith: "I believe because it is absurd". It may not be far off the day when the mentalist view will come to be seen as creationism is now regarded.

A physicalist faced with the same challenge would say: although not available to our introspective stance, all that runs through our minds - the cornucopia of subjective life - has concrete, objective causes and is the product of observable neurophysiological processes which can be analysed.

Our subjective states coexist with the objective changes in the brain, but this does not imply that they play any explanatory role. It is illusory to take as a cause that which rises to consciousness like an act of will or intention to act. Subjective experience is the last breath in the chain of neural events which precede it, like a flutter produced by the flapping of a flock of birds - rustling is the reverberation of the flyover. Mental events which encapsulate our conscious and unconscious life (such as dreams) are effects which have yet to be explained, but devoided of causal efficacy.

A mental state ("I must eat") is never really produced by another mental state ("I am feeling hungry"); they are both produced by states of the brain. When a thought seems to evoke another by association, it is not really a case of one thought which draws or attracts another thought - the association does not occur between the two thoughts but rather between the brain states or particular neurophysiological configurations underlying these thoughts.

One of these states of the brain brings forth another, making itself accompanied, in its course, by the particular mental state it produces. The fulfilment of the act by the body's muscles ("fork to mouth") and the digestion regulated by the hypothalamus crown the process. In short, the mental intermediary is a redundant byproduct and surface phenomenon - epiphenomenon - in relation to the autonomous functioning of the physical organism.

**IX - THE JIGSAW OF THE MIND-BRAIN PUZZLE IS** far from complete - many key pieces are still missing. But the overall picture and the direction of the significant advances so far attained, particularly over the last twenty years, seem pretty clear.

All the spearheads of scientific research are honed for the same target. The more we delve into the knowledge of the secrets of the black-box, the more unavoidable the "astonishing hypothesis" (Francis Crick) becomes, and the greater the plausibility of the disconcerting conclusion that *one's mental states stand in relation to one's brain as the whistle of a kettle stands in relation to its working mechanism.*

In contrast to what our intuitive psychology would lead us to believe, it is not the whistle which makes the water boil. but rather, it is the boiling water that makes the whistle blow, as research in neuroscience and related fields has been step by step spelling out with ever greater detail and accuracy.

If this is true, then our subjective and inner experience of life is nothing but a fanciful and intriguing byproduct of physical processes - thus the term *physicalism*, instead of the traditional, albeit imprecise* materialism* - which occur in an closed, autonomic and self-sufficient manner in the organism; although endowed with unlimited richness and fascination, our mental states would turn out to be entirely innocuous and devoid of causal power over the objective physical world to which they belong.

Though we have a familiar and deeply-rooted feeling of control over our thoughts and actions, this sensation is again no more than a deceiving fancy of our brain; a mere illusion leftover from the pre-scientific, archaic era in which the belief prevailed that every living and moving thing in nature had a soul.

Physicalism subverts our intuitive psychology and casts a disturbing light on all we are used to think about the human condition in the natural world. It was no coincidence that La Mettrie, physician and philosopher, author of *L'homme machine*, the seminal and bold physicalist manifesto of the XVIII century, achieved the feat of making all the religions of Europe unite against him, no matter how fiercely they fought otherwise against one another.

The idea itself is awesome yet a syllogism suffices to summarize it. The laws and regularities governing the world are independent of my will (greater premise); my will is the product of the same laws and regularities which govern the world (lesser premise); therefore my will is independent of my will (conclusion). If the premises hold true then the conclusion is inevitable.
